# Radiation exposure awareness from patients undergoing nuclear medicine diagnostic ^99m^Tc-MDP bone scans and 2-deoxy-2-(18F) fluoro-D-glucose PET/computed tomography scans

**DOI:** 10.1097/MNM.0000000000001177

**Published:** 2020-03-13

**Authors:** Ana S.F. Ribeiro, Olga Husson, Nicholas Drey, Iain Murray, Katherine May, Jim Thurston, Wim J.G. Oyen

**Affiliations:** a The Royal Marsden Hospital NHS Foundation Trust; b Institute of Cancer Research, Sutton; c City, University of London, London, UK

**Keywords:** awareness, ionising radiation, nuclear medicine, patient engagement

## Abstract

Medical imaging is on average the largest source of artificial radiation exposure worldwide. This study seeks to understand patient’s awareness of radiation exposure derived from nuclear medicine diagnostic scans and assess if current information provided by leaflets is adequate.

**Methods:**

Single-centre cross-sectional questionnaire study applied to bone scan and FDG PET/computed tomography patients, at a nuclear medicine and PET/computed tomography department over a 15-week period in 2018. Questionnaires on dose comparators were designed in collaboration with patients, public, and experts in radiation exposure. Qualitative data were analysed using thematic analysis and quantitative data using SPSS (V. 24).

**Results:**

A total of 102 questionnaires were completed (bone scan = 50; FDG PET/computed tomography = 52). Across both groups, 33/102 (32.4%) patients reported having a reasonable understanding of nuclear medicine and 21/102 (20.6%) reported a reasonable knowledge of ionising radiations. When asked to compare the exposure dose of respective scans with common comparators 8/50 (16%) of bone scan patients and 11/52 (21.2%) FDG PET/computed tomography answered correctly. On leaflet information, 15/85 (17.6%) patients reported the leaflets do not provide enough information on radiation exposure and of these 10/15 (66.7%) commented the leaflets should incorporate more information on radiation exposure dose.

**Conclusion:**

More observational and qualitative studies in collaboration with patients are warranted to evaluate patients’ understanding and preferences in communication of radiation exposure from nuclear medicine imaging. This will ensure communication tools and guidelines developed to comply with ionising radiation (medical exposure) regulation 2017 are according to patients needs and preferences.

## Abstract 2

Supplemental Digital Content is available in the text.

## Introduction

Worldwide, medical exposure is the highest source of artificial radiation, and after natural sources are the second-largest source of exposure, accounting for 20% of the total exposure [1]. In nuclear medicine imaging, some inherent risks derive from exposure to the ionising radiation that these techniques use, however, these risks are balanced or outweighed by the benefits to health of the individual according to the two principles in radiation protection, justification, and optimisation [2]. Justification ensures the procedure is necessary, justifiable, and the benefits outweigh the risks. Optimisation ensures that all practical measures are in place to reduce unnecessary exposure and risk to both the patient and the operator.

Informing patients of the levels of ionising radiation exposure associated with medical imaging procedures together with the risks and benefits is fundamental to comply with current UK legislation as per ionising radiation (medical exposure) regulations 2017 (IR(ME)R 2017)[3] but also to enable patient decision-making, improve acceptance of diagnostic scans and confidence in the medical care received.

Describing and explaining radiation exposure and associated risks can, however, be challenging. This is mainly due to patient’s background knowledge of radiation and health literacy levels that can vary greatly. The information presented to patients may not be meaningful [2]. Detailed information on radiation exposure from diagnostic imaging can also lead to unwanted effects such as increased anxiety levels in patients who request additional information [4].

Lack of communication on radiation exposure can also derive from lack of awareness and knowledge from healthcare professionals, including physicians, radiographers, and nuclear medicine technologists. The results of recent studies results[5–10] and a systematic review of 14 peer-reviewed articles in 2013 also demonstrated a lack of physician’s knowledge and a tendency to underestimate ionising radiation exposure from medical imaging [9].

Our recent review conducted to identify existing literature on radiation exposure awareness of patients when undergoing nuclear medicine diagnostic scans concluded that across medical imaging there is a lack of knowledge from patients. In addition, there is a general underestimation from health professionals on ionising radiation exposure with a need for improvement in communication between professionals and patients [11].

The main objective of this study was to establish if patients understand how much radiation they are exposed to when they undergo two of the most common nuclear medicine procedures performed in the department where this study was conducted and if current leaflets provide adequate information. The procedures are bone scans and 2-deoxy-2-(18F) fluoro-D-glucose (^18^F-FDG) PET/computed tomography scans (PET/CT). The results aim to influence the modification of information leaflets and improve communication between healthcare professionals and patients regarding ionising radiation in medical imaging.

## Methods

This cross-sectional questionnaire study was performed at the Royal Marsden NHS Foundation Trust. The study was conducted in accordance with HRA approval processes. REC approval was received on 30 January 2018 (REC reference 18/NS/0008) and HRA approval (IRAS project ID 233911) was received on 6 February 2018.

### Questionnaire

A review of existing validated questionnaires did not find a suitable tool. A detailed questionnaire to identify prior knowledge in nuclear medicine and of ionising radiation used in nuclear medicine by comparing the diagnostic procedures with common comparators[12,13] was developed iteratively in collaboration with co-investigators, three patient representatives, a Radiation Protection Adviser, an epidemiologist and a physicist. The questionnaire was tested within the institutional nuclear medicine department, which included technologists, radiographers, specialist nurses, and physicists. Modified questionnaires for radiation awareness comparators were designed according to groups. Group 1: patients attending for a ^99m^Tc-MDP bone scan; group 2: patients attending for an ^18^F-FDG PET/CT. Questionnaires had a brief introduction followed by four sections, (a) demographic questions (sex, age, education, and health literacy); (b) patient self-reported knowledge (e.g. *Do you understand how nuclear medicine is used in medical diagnosis?*); (c) patient awareness, with four questions that compared the doses of radiation from the two diagnostic scans to natural background radiation, chest X-rays, CT scans, and transatlantic flights (e.g. *What do you think a bone scan is equivalent to in terms of natural background radiation?*); and (d) two open-ended questions on current suitability of patient information leaflets and further comments, in order to complement and enrich the quantitative data collected. Full questionnaires can be seen in Supplementary Appendix 1, Supplemental digital content 1, http://links.lww.com/NMC/A164 and leaflet content in Supplementary Appendix 2, Supplemental digital content 2, http://links.lww.com/NMC/A165.

### Participants

Purposive sampling was applied, with participants over the age of 18 recruited from a Nuclear Medicine Department that performs a variety of diagnostic and therapeutic procedures, mainly to an oncological population. This descriptive study did not test any hypotheses and so no formal sample size calculations were performed. With 50 participants in each of groups 1 and 2, proportions within each group can be reported with 95% confidence intervals of not more than ± 14%. Recruitment took place from 26 February 2018 to 6 June 2018.

### Data analysis

#### Quantitative data

Data were analysed using IBM SPSS Statistics 24. Responses to questionnaires were reported using summary descriptive statistics with assessment of differences between groups performed using the Chi-square tests (and Fisher’s exact) for categorical variables and Mann–Whitney *U* test for non-parametric scale/ordinal variables, assuming significance at *P* < 0.05. Missing data checked using IBM SPSS Missing Value Analysis.

#### Qualitative data

Data from the two open-ended questions were analysed using the model proposed by Braun and Clarke [14]. This systematic and rigorous method involves six phases, resulting in final themes that describe the data and are supported by extracts of patient’s comments and views. The first question aimed to capture more in depth information on patient’s views of the leaflet, the second question was general and broader where patients could express any additional views, comments or concerns, as typically included in questionnaires.

## Results

### Quantitative data

A total of 102 completed questionnaires with G1 n = 50 and G2 n = 52 were collected and analysed. Data distribution for G1, G2, and combined G1 and G2 were not normally distributed, and variable age was missing for 11 cases (10.8%) and education for two cases (2%). Patient characteristics are presented in Table 1.

**Table 1:**
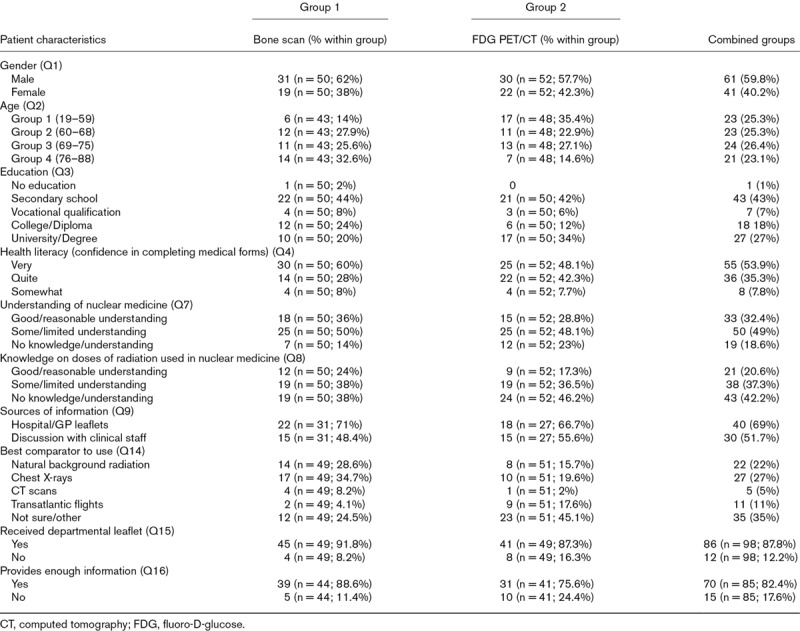
Patient characteristics

Within G1 31/50 (62%) patients were male and 19/50 (38%) were female with a median age of 72 (range: 63–80). Half the patients reported having some or limited understanding of nuclear medicine and 19/50 (38%) some knowledge of radiation doses used in nuclear medicine. The main sources of information for patients were through hospital/GP leaflets 22/31 (71%), followed by discussion with medical staff 15/31 (48.4%). The best comparator for doses of radiation used in bone scans were, chest X-rays 17/49 (34.7%) followed by natural background radiation 14/49 (28.6%), while 12/49 (24.5%) patients were not sure on the best comparator to use.

G2 had 30/52 (57.7%) male and 22/52 (42.3%) female patients, with a median age of 66 (range: 46–72). Approximately half the patients 25/52 (48%) reported some understanding of nuclear medicine and 19/52 (37%) some understanding in radiation doses used in nuclear medicine. Patients mainly received information from hospital/GP 18/27 (66.7%) and 15/27 (55.6%) via discussion with medical staff. As to preferences for best comparator to use, 23/51 (45.1%) patients reported not being sure of the best comparator to use with a patient adding under other: ‘the more information the better,’ followed by 10/51 (19.6%) patients preferring chest X-rays as a comparator.

**Fig. 1:**
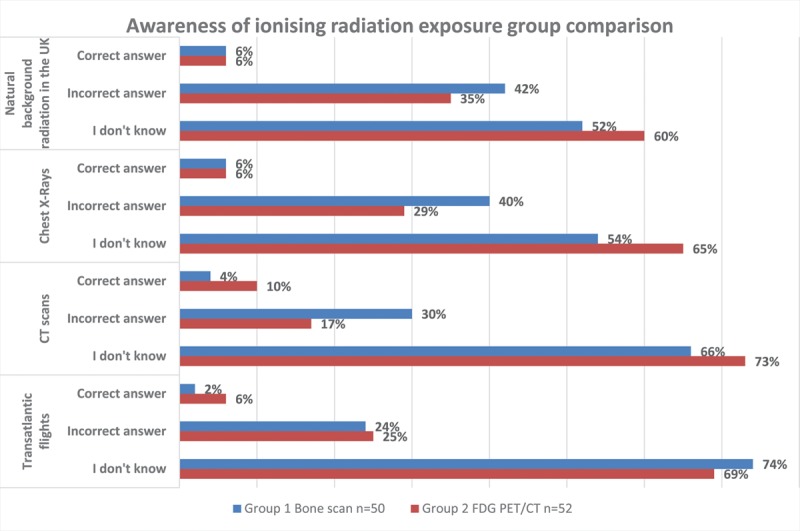
Group comparison on awareness of radiation exposure.

Patient awareness and understanding of exposure to ionising radiation derived from bone scans and FDG PET/CT scans were assessed in section (c) (Q10-Q13) (Fig. 1). Across all four questions, 8/50 (16%) of patients from G1 answered correctly with 9/200 (4.5%) correct answers. The majority of patients answered, ‘I don’t know’ 123/200 (61.5%), followed by incorrect answers 68/200 (34%). All patients who answered correctly received information from hospital/GP leaflets and through discussion with medical staff. The majority of patients 45/49 (91.8%) received the departmental leaflets and the majority 39/44 (88.6%) agreed it provided enough information regarding doses of radiation used in nuclear medicine. Within G2 11/52 (21.2%) patients answered correctly with 14/200 (6.7%) correct answers, 55/200 (26.4%) incorrect answers and the vast majority answered, ‘I don’t know’ 139/200 (66.8%). Patients who had previous scans gave more correct answers 8/31 (25.8%) when compared to patients who did not have previous scans, 3/21 (14.3%). The main sources of information for patients who answered correctly were hospital/GP leaflets, followed by discussion with medical staff and medical/science websites. The majority of patients from G2 received the departmental leaflets 41/49 (87.3%) and thought the leaflets provided enough information 31/41 (75.6%). Patients with higher educational status answered correctly to a higher number of questions, with 5 patients in G1 and 13 patients in G2.

### Qualitative data

In total, there were 55 comments across the two questions, 10 relating to adequateness of the leaflet and 45 relating to other issues, 14 comments were deemed not relevant, as these mainly addressed hospital facilities.

Data were reviewed twice, coded, and basic themes were further analysed and merged into higher themes: (a) adequate information and (b) communication and trust in the clinical team. From these, the global theme: communication and information are essential to improve patient overall experience when undertaking a nuclear medicine diagnostic scan was identified.

#### Adequate information

Patients commented on the need for more information regarding ionising radiation used in diagnostic procedures using common comparators such as the ones used in the questionnaire. For example: ‘Leaflet doesn’t say much about the level of radiation at all’, with another patient’s comment ‘it is not just bone scans but other scans, X-rays, therapy that is relevant on a cumulative basis to an individual measured against a “safe” dose.’

The information, however, must be meaningful to patients, ‘too much knowledge can frighten people…,’ ‘reference to nuclear radiation is not likely to cause patients to feel confident in treatment being harmless,’ and ‘knowing a level is not really meaningful on its own.’ Some patients indicated that they would not require more information ‘I don’t believe I would benefit from a more detailed explanation of radiation doses. I would go ahead regardless as the scan is important for my treatment.’

#### Communication and trust in the clinical team

This was well illustrated by patients’ comments: ‘whilst more information is always useful I am happy to follow medical advice that a scan is necessary’; ‘I feel reassured by the verbal explanation from the staff’ and ‘I have complete trust in the medical staff and assume the treatment they decide on is the best suited to my needs’; to ‘keep up the communication between patient and yourselves. It will continue to improve general understanding of issues.’

##### Global theme: communication and information are essential to improve patient overall experience when undertaking a nuclear medicine diagnostic scan

Communication and information are two fundamental aspects expressed by patients, so that they are able to understand the procedures they have been referred for. While some patients would like to receive more information on radiation exposure derived not only from nuclear medicine scans but also from other imaging procedures, for other patients complete trust in the health professionals means they do not necessarily appreciate more information and for some unless it is meaningful it can cause distress and fear.

## Discussion

This study aimed to establish if patients understand how much radiation they are exposed to when they have a bone scan or a PET/CT with a view to modify existing patient leaflets. The quantitative results from the four questions on awareness of radiation exposure in section (c) include a low number of correct answers alongside a high number of ‘I don’t know,’ across both groups. These findings are in accordance with studies included in the literature review, all of which reported patients lack awareness and knowledge on ionising radiation exposure [15–17]. Similar findings were also reported in a recent mixed methods study comprising a survey and focus groups conducted in Spain aimed at evaluating general population understanding of the benefits and risks associated with five imaging modalities (nuclear medicine not included) as well as their opinions on how that information should be delivered [18]. Results demonstrated the general population lacked information concerning ionising radiation exposure from medical imaging and more information should be provided to patients to make them aware of the radiation exposure when undergoing scans involving ionising radiation. A study in 2011 by Freudenberg and Beyer[19] concluded that experts within nuclear medicine and radiology should aim to educate and inform not only patients but also referring clinicians who also lack knowledge.

The qualitative data, resulting from the two open-ended questions, however, highlights a mixed response. Some patients state they would not benefit from more detailed information and indicate that they trust the clinical team, whereas others suggest communication between healthcare professionals and patients should be encouraged. Patients also suggested incorporating more meaningful information on leaflets such as comparisons of risk and benefits of the associated exposures from the diagnostic tests. This is particularly relevant since across both groups the majority of patients received information on radiation exposure from nuclear medicine imaging via hospital leaflets.

The results from section 3 of the questionnaire demonstrate a low awareness of radiation exposure from diagnostic imaging when put side-to-side with common comparators. As this study illustrates, however, this might not be the correct way to present data to patients. Several comparators have historically been used to describe the dose of radiation and associated risks, such as natural background radiation, flights, other clinical risks, and rate of cancer[20] as a means to simplify and inform patients and public. However, on their own, they may not fully convey the information to the general population and the perception of risk is different among individuals, just as for each patient, the risk/benefit ratio will be different. As per a patient comment, the comparison to a ‘safe dose’ is also important, as the exposure on its own can be meaningless.

The recent changes in the UK, with IRMER 2017, Schedule 2–Employers Procedures, state that patients must be informed ‘(i) providing that wherever practicable, and prior to an exposure taking place, the patient or their representative is provided with adequate information relating to the benefits and risks associated with the radiation dose from the exposure’ [3].

Imaging departments are required to provide clear information about radiation exposure to patients, and there is the need for better communication about the risks and benefits to patients. Describing the benefits of having a particular scan/therapy that uses ionising radiation by demonstrating the potential risk of not having the scan/therapy is another possible way to convey information to patients and in some cases perhaps more meaningful [21,22].

Continuous professional education and development for health professionals are also essential to ensure the best care is provided and professionals are able to correctly inform patients and relatives [19,23–25]. A UK study of radiographers’ perspectives on CT risks and how information is given to patients highlighted that radiographers often fail to communicate effectively with patients when it comes to ionising radiation exposure. This was mainly due to the fear of discouraging patients from undergoing the scans, time constraints with busy clinics and a lack of knowledge on radiation exposure and how best to explain it [26]. To truly reflect a shared decision-making process where clinicians actively engage with patients for patient-centred care, it is crucial that healthcare professionals understand and are able to inform patients on the benefits and potential risks of ionising radiation for medical purposes supported by best evidence-based practice and guidelines [19,24,27].

This study and questionnaire are based on the accepted linear no-threshold (LNT) model as the impact of alternative models remains unclear. LNT model has been in practice for the last 70 years, and it was introduced as a mean to simplify radiation protection [28]. It is based on the fact that any exposure to ionising radiation, including very low dose such as X-ray, can lead to carcinogenesis’s and the risk is proportional to the dose—that is, double the radiation exposure dose means double the risk.

Although there is a growing body of literature against the LNT, it remains the model underpinning current legislative frameworks and therefore was the basis for this study [29–31].

### Recommendation for future research

Engaging with patient and public involvement and engagement in wider research to assess knowledge, develop, and promote education is essential to improve patient and public awareness and knowledge. Good communication between healthcare professionals and patients on ionising radiation has the ability to not only increase patient knowledge but also potentially reduce anxiety and empower patients to have confidence in their treatment decisions.

### Conclusion

This study aimed to provide a current status of patient awareness in this particular clinical setting in view of current legislation modifications with the revised IR(ME)R regulations and the need to better inform patients.

The quantitative results demonstrate limited patient awareness on radiation exposure. The qualitative results, however, indicate that this is not necessarily an important factor for patients themselves. Patients would welcome accurate and clear information they can easily understand regarding the risks and benefits derived from medical imaging in general.

In order to comply with recent changes in UK legislation with IR(ME)R 2017. It is crucial to develop communication guidelines in close collaboration with patients and public in order to provide patients with accurate information that they can relate to and thus ensure the principle of informed consent is present.

## Limitations

A review of existing validated questionnaires did not find a suitable tool. A questionnaire was developed solely for this study, and therefore, we do not recommend this to be used in other studies. We recommend that whenever possible a validated questionnaire is used.

The study involved a relatively small sample from a single department, with no significances in the statistical tests performed and limited qualitative data. Although cautious interpretation is required when extrapolating the study results to a wider population or when identifying potential factors that affect patient awareness and understanding, cross tabulation using frequencies and percentages is valid for the population in question, with results being similar to the studies included in literature review.

## Acknowledgements

We acknowledge the support of the NIHR Biomedical Research Centre and the NIHR Clinical Research Facility at The Royal Marsden and Institute of Cancer Research.

ClinicalTrials.gov Identifier: NCT03458585.

## Conflicts of interest

There are no conflicts of interest.

## Supplementary Material
