# Supplementary material for: Radiation exposure awareness from patients undergoing nuclear medicine diagnostic 99mTc-MDP bone scans and 2-deoxy-2-(18F) fluoro-D-glucose PET/computed tomography scans
Source: Nucl Med Commun. 2020 Mar 13;41(6):582–8. doi: 10.1097/MNM.0000000000001177 (PMC7242175; doi:10.1097/MNM.0000000000001177)
Supplement: Supplementary file 1 [file nmc-41-582-s001.pdf]

(Appendix 1)

Radiation **E**xposure **A**wareness from patients undergoing nuclear medicine diagnostic scans

REA STUDY

Group 1– Patients having a Bone scan

Date of completion (today): ______ (day)/______ (month)/______ (year)

Nuclear medicine uses small amounts of radioactive materials, called radiopharmaceuticals or radiotracers, to diagnose and treat disease. For a bone scan we use ^{99m} Technitium - Methylene Diphosphonate (^{99m}Tc- MDP).

Nuclear Medicine scans such as the Bone scan that you had allow us to obtain excellent and unique information about your bones.

What is radiation?

Radiation is simply a type of energy. The most familiar form of radiation is visible light, like that produced from the sun or a light bulb.

Nuclear Medicine uses a type of radiation called Ionising radiation to image your body and for the bone scan it uses gamma-rays which are captured by the gamma-camera and create your scan.

This questionnaire is aimed at finding out what is the current knowledge of patients on radiation exposure from Nuclear Medicine diagnostic scans and if current information provided to patients is sufficient to allow them to understand what the radiation dose from that scan is.

First we would like to ask you some general demographic questions

SECTION 1: ABOUT YOU

1. What is your gender?

❑ Male
❑ Female
❑ Prefer not to say

2. What is your age?

______________/Prefer not to say

3. Which of the following best describes your highest level of education completed?
❑ No education or primary school
❑ Secondary school
❑ Vocational qualification
❑ College/Diploma
❑ University/Degree
❑ Other (please specify) _______________________________________

4. How confident are you filling out medical forms by yourself?
❑ Very
❑ Quite
❑ Somewhat
❑ A little
❑ Not at all

5. Have you had a Bone scan before?
❑ Yes
❑ No → continue to question 7

6. How many bone scans have you had before?

❑ 1
❑ 2
❑ 3 or more

SECTION 2: UNDERSTANDING OF NUCLEAR MEDICINE

7. Do you understand how nuclear medicine is used in medical diagnosis?

❑ I have a good or a reasonable understanding

❑ I have some or a limited understanding

❑ I do not understand how it works

8. Do you have any knowledge about the doses of radiation used in nuclear medicine?

❑ I have a good or a reasonable understanding

❑ I have some or a limited understanding

❑ I have no prior knowledge or understanding

9. If you ticked either of the first two options in Q8 how did you get your information about it? Please tick all that apply

❑ Hospital leaflets

❑ Through discussion with clinical staff at hospital e.g. Doctor, Nurse, Radiographer

❑ Printed and broadcast media, e.g. Newspapers, magazines, TV, radio

❑ Social media online, e.g. Twitter, Facebook, Reddit

❑Medical and science related websites on the internet

❑Family/friends

❑Other: ___________________________________

SECTION 3: AWARENESS OF RADIATION EXPOSURE

A good way to explain the radiation exposure from nuclear medicine procedures is to compare it other sources of exposure such as natural background radiation, chest X-rays, CT scans and flights.

Background radiation is present in the environment and originates from a variety of sources both natural and artificial, such as cosmic rays, rocks and atmosphere and in certain areas it can also derive from nuclear weapons testing and nuclear accidents.

10. What do you think a Bone scan is equivalent to in terms of natural background radiation in the UK?

❑ 2 Days
❑ 2 Years
❑ 20 Years
❑ 200 Years
❑ I don't know

Nuclear medicine scans use a different type of radiation to X-rays; however it is possible to compare them in terms of exposure to the patient. The most common procedure for X-rays is a Chest X-Ray, which is a relatively low dose imaging procedure.

11. A Bone scan is equivalent to how many Chest X-Rays?

❑ 3 Chest X-Ray
❑ 30 Chest X-Rays
❑ 300 Chest X-Rays
❑ 3000 Chest X-Rays
❑ I don't know

Computerized Tomography (CT) of Thorax, Abdomen and Pelvis (TAP) with contrast enhanced (with either oral or IV contrast) is one of the most common CT procedures.

12. A Bone scan is equivalent to how many CT scan(s)?

❑ A quarter (1/4) of a CT scan
❑ 1 CT scan
❑ 10 CT scans
❑ 100 CT scans
❑ I don’t know

Another good way to communicate radiation exposure to patients is to compare it to the approximate equivalent in terms of everyday tasks such as:

Transatlantic flights: During transatlantic flights you are exposed to higher levels of cosmic radiation due to flying at higher altitudes.

13. A bone scan is equivalent to how many transatlantic flights?

❑ 5 Flights
❑ 50 Flights
❑ 500 Flights
❑ 5000 Flights
❑ I don’t know

14. Based on the above questions what do you think would be the best comparator to use to explain radiation exposure to patients having nuclear medicine scans?

❑ Natural background radiation
❑ Chest X-Rays

❑ CT
❑ Transatlantic flights
❑ Note sure/other: ______________________________

15. Have you received our departmental leaflet explaining what a Bone scan procedure involves?

❑ Yes
❑ No → continue to question 18

16. If you have received it do you think it provided you with enough information regarding doses of radiation used in the Bone scan?

❑ Yes → continue to question 18
❑ No

SECTION 4: FINAL COMMENTS

17. If No what else do you think could be improved?

18. Is there anything else you would like to tell us?

Thank you for taking the time to complete this questionnaire

(Appendix 1)

<u>**R**adiation **E**xposure **A**wareness from patients undergoing nuclear medicine diagnostic scans</u>

REA STUDY

Group 2– Patients having FDG PET/CT scan

Date of completion (today): ______ (day)/______ (month)/______(year)

Nuclear medicine uses small amounts of radioactive materials, called radiopharmaceuticals or radiotracers to diagnose and treat disease. For a FDG PET/CT scan we use 2-Deoxy-2-[^{18}F]fluoroglucose (^{18}F-FDG)

Nuclear Medicine scans such as the FDG PET/CT scan you had allow us to obtain excellent and unique information on how for example tumours behave.

<u>What is radiation?</u>

Radiation is simply a type of energy and the most familiar form of radiation is visible light, like that produced from the sun or a light bulb.

Nuclear Medicine uses a type of radiation called Ionising radiation to image your body and for the FDG PET/CT you just had it uses positive electrons (positrons)that interact within your body, are captured by the PET/CT scanner and create your scan. This is possible due to the radioactive (^{18}F is the radioactive component) injection you received which is labelled with a modified sugar (FDG) that binds to tumours.

This questionnaire is aimed at finding out what is the current knowledge of patients on radiation exposure from Nuclear Medicine diagnostic scans and if current information provided to patients is sufficient to allow them to understand what the radiation dose from that scan is.

First we would like to ask you some general demographic questions

SECTION 1: ABOUT YOU

1. What is your gender?

❑ Male

❑ Female

❑ Prefer not to say

2. What is your age?

_______________/Prefer not to say

3. Which of the following best describes your highest level of education completed?

❑ No education or primary school

❑ Secondary school

❑ Vocational qualification

❑ College/Diploma

❑ University/Degree

❑ Other (please specify) ____

4. How confident are you filling out medical forms by yourself?

❑ Very

❑ Quite

❑ Somewhat

❑ A little

❑ Not at all

5. Have you had an ^{18}F-FDG PET/CT scan before?

❑ Yes

❑ No → continue to question 7

6. How many scans have you had before?

❑ 1

❑ 2

❑ 3 or more

SECTION 2: UNDERSTANDING OF NUCLEAR MEDICINE

7. Do you understand how nuclear medicine is used in medical diagnosis?

❑ I have a good or a reasonable understanding

❑ I have some or a limited understanding

❑ I do not understand how it works

8. Do you have any knowledge about the doses of radiation used in nuclear medicine?

❑ I have a good or a reasonable understanding

❑ I have some or a limited understanding

❑ I have no prior knowledge or understanding

9. If you ticked either of the first two options in Q8 how did you get your information about it? Please tick all that apply

❑ Hospital/GP leaflets

❑ Through discussion with clinical staff at hospital e.g. Doctor, Nurse, Radiographer

❑ Printed and broadcast media, e.g. Newspapers, magazines, TV, radio

❑ Social media online, e.g. Twitter, Facebook, Reddit

❑Medical and science related websites on the internet

❑Family/friends

❑Other: ________________________________

SECTION 3: AWARENESS OF RADIATION EXPOSURE

A good way to explain the radiation exposure from nuclear medicine procedures is to compare it to other sources of exposure such as natural background radiation, chest X-rays, flights and dental X-rays.

Natural background radiation is present in the environment and originates from a variety of sources both natural and artificial, such as cosmic rays, rocks and atmosphere and in certain areas it can also derive from nuclear weapons testing and nuclear accidents.

10. What do you think an ^{18}F-FDG PET/CT scan is equivalent to in terms of natural background radiation in the UK?

❑ 6 months
❑ 6 Years
❑ 60 Years
❑ 600 Years
❑ I don't know

Nuclear medicine scans use a different type of radiation to X-rays; however it is possible to compare them in terms of exposure to the patient. The most common procedure for X-rays is a Chest X-Ray, which is a relatively low dose imaging procedure.

11. An ^{18}F-FDG PET/CT is equivalent to how many Chest X-Rays?

❑ 10 Chest X-Rays
❑ 100 Chest X-Rays
❑ 1000 Chest X-Rays
❑ 10,000 Chest X-Rays
❑ I don't know

Computerized Tomography (CT) of Thorax, Abdomen and Pelvis (TAP) with contrast enhanced (with either oral or IV contrast) is one of the most common CT procedures.

12. An ^{18}F-FDG PET/CT scan is equivalent to how many CT scan(s)?

❑ Half (1/2) of a CT scan
❑ One and a half (1 ½) CT scan
❑ 10 CT scans
❑ 100 CT scans
❑ I don't know

A good way to communicate dose to patients is to compare it to the approximate equivalent in terms of everyday tasks such as:

Transatlantic flights: During transatlantic flights you are exposed to higher levels of cosmic radiation due to flying at higher altitudes.

13. An ^{18}F-FDG PET/CT scan is equivalent to how many transatlantic flights?

❑ 20 Flights
❑ 200 Flights
❑ 2000 Flights
❑ 20,000 Flights
❑ I don't know

14. Based on the above questions what do you think would be the best comparator to use to explain radiation exposure to patients having nuclear medicine scans?

❑ Natural background radiation
❑ Chest X-Rays
❑ CT
❑ Transatlantic flights
❑ Note sure/other: ______________________________

15. Have you received our departmental leaflet explaining what ^{18}F-FDG PET/CT scan procedure involves?

❑ Yes
❑ No → continue to question 18

16. If you have received it do you think it provided you with enough information regarding doses of radiation used in the ^{18}F-FDG PET/CT scan?

❑ Yes → continue to question 18
❑ No

SECTION 4: FINAL COMMENTS

17. If No what else do you think could be improved?

18. Is there anything else you would like to tell us?

Thank you for taking the time to complete this questionnaire
