# Supplementary material for: Radiation exposure awareness from patients undergoing nuclear medicine diagnostic 99mTc-MDP bone scans and 2-deoxy-2-(18F) fluoro-D-glucose PET/computed tomography scans
Source: Nucl Med Commun. 2020 Mar 13;41(6):582–8. doi: 10.1097/MNM.0000000000001177 (PMC7242175; doi:10.1097/MNM.0000000000001177)
Supplement: Supplementary file 2 [file nmc-41-582-s002.pdf]

(Appendix 2)

Introduction

Your doctor has recommended that you have a bone scan. This is a nuclear medicine test. A scan of your skeleton is taken to help your doctor find out if there are any problems in your bones.

What is nuclear medicine?

Nuclear medicine helps doctors to check how well different parts of your body are working. A small amount of a radioactive substance (tracer) is given, usually by injection into a vein. The tracer gives off gamma rays, so we can measure the distribution of that tracer in your body. This measurement is usually done using a gamma camera, although occasionally measurement of blood samples may be needed.

Is there any risk from the radiation?

The amount of radiation involved is small, and is similar to the amount used in some x-ray procedures. The benefits of the scan far outweigh any possible risk from the radiation. Your referring doctor will have considered this carefully before asking us to carry out this examination. **However, it is essential that you read the 'Important points' section below. If you have any concerns please contact us.**

What preparation do I need for my bone scan?

There is no special preparation for your scan. You can eat and drink as usual. You do not need to have a full bladder.

You can continue to take any medication that has been prescribed for you by your doctor. It is also safe for you to take "over the counter" medicines.

How is my bone scan carried out?

On arrival in the department, we will give you a small injection of radioactive tracer into a vein. This will be absorbed into your bones over about three hours. During this time you can leave the department but you will need to drink at least two litres of fluid (tea, coffee, water etc). However, you do not need a full bladder for your scan.

Occasionally it may be necessary to take scans while the tracer is being injected in which case you will be asked to lie
or sit in front of the gamma camera. These scans take about 10 minutes. You will be asked to take off your shoes and remove jewellery and metal items from your pockets. There is no need to take off your clothes for the scan but if any of your clothing has metal studs or buttons, you may be asked to remove it. When
you return to have your scans taken three hours later, you will be asked to lie or sit in front of the gamma camera. The scan will take about 30-45 minutes.

Are there any side effects?

The tracer that we inject will not produce any side effects. You can continue with your usual daily activities. In particular it will not make you drowsy and so will not prevent you from driving a car.

What happens after my bone scan?

Once the scan is completed you will be able to leave the department immediately. You will be able to eat
and drink what you like. You may go anywhere you wish but you should avoid prolonged close contact with children for the rest of the day. This is to avoid exposing children to unnecessary radiation.

How will I get the results of my bone scan?

Your bone scan will be reported on by the nuclear medicine consultant within 48 hours of

completion. The results of your test will then be made available to the doctor who referred you

Important points

- Due to the nature of these investigations we advise that **you should not be accompanied by anyone who is pregnant and should not bring young children to the department.** With most of these investigations the level of radioactivity will have decreased to a safe level by the time you arrive home after the scan. However, some investigations need more specific restrictions with regards to contact with young children and these will be explained.
- If you are afraid of needles you can ask for Ametop cream/spray. This is a local anaesthetic, which is placed on the skin half an hour before the injection. Please ring the Nuclear Medicine Department if you would like this.
- If you are pregnant or breastfeeding please contact the department as soon as possible to find out if you can have this test. Generally, nuclear medicine tests are not carried out on pregnant women unless absolutely necessary and then the dose of radioactivity will probably be reduced.

(Appendix 2)

Introduction

Your doctor has recommended that you have an FDG PET/CT scan. This is a nuclear medicine test combining a PET scan and a CT scan.

What is positron emission tomography (PET)?

PET is a medical imaging technique in which a radioactive tracer is injected into a vein. The most commonly used tracer in PET is FDG (fluoro-deoxy- glucose), which is a radioactive form of glucose. The scan shows how the body breaks down and uses glucose.
Abnormal cells use glucose differently and this will show up on the scan. This radioactive tracer has a short shelf life and is made on the day of the scan. Appointments may be cancelled at short notice if there are tracer production problems.

What is CT?

A CT (Computerised Tomography) scan uses x-rays to produce images of the body. By combining PET and CT, we are able to provide important information about many conditions affecting the different organs of your body. This will help your doctor to plan appropriate treatment for you.

Is there any risk from the radiation?

The amount of radiation involved is small, and is similar to the amount used in some x-ray procedures. The benefits of the scan far outweigh any possible risk from the radiation. Your referring doctor will have considered this carefully before asking us to carry out this examination. However, please read the *Important points* section below. If you have any concerns, please contact us.

What preparation do I need for my FDG PET/CT scan?

Do not have anything to eat for **six hours** before your appointment. During this time you can drink as much still/tap water as you like. You do not need to have a full bladder for your scan.

Once your appointment has been confirmed, it is essential that you contact the Nuclear Medicine Department to inform us about any medication that you may be taking and if you have diabetes. It is safe for you to take ‘over the counter’ medicines.

How is a FDG PET/CT scan carried out?

When you arrive at the department, we will ask you to change into a hospital gown and remove all jewellery. You will then be taken to a preparation room to lie on a couch, and have a small plastic tube (cannula) inserted into a vein in your arm. We will then give you a small injection of a

radioactive tracer and ask you to remain lying down for about one hour before your scan.

After one hour, we will ask you to move into the scanning room and onto the scanning bed. The scan will take about 30 to 90 minutes. You will be scanned with your arms raised above your head. Occasionally we may also scan you with your arms by your sides.

Are there any side effects?

The tracer that we inject will not produce any side effects. You can continue with your usual daily activities. In particular, it will not make you drowsy and so will not prevent you from driving.

What happens after my FDG PET/CT scan?

Once the scan is completed, you will be able to leave the department immediately. You will be able to eat
and drink what you like. You may go anywhere you wish but you should avoid prolonged close contact with children for the rest of the day. This is to avoid exposing children to unnecessary radiation.

How will I get the results of my FDG PET/CT scan?

Your FDG PET/CT scan will be reported on by the nuclear medicine consultant within 24 hours of completion. The results of your test will then be made available to the doctor who referred you.

Important points

Due to the nature of these investigations we advise that **you should not be accompanied by anyone who is pregnant and should not bring young children to the department.** With most of these investigations, the level of radioactivity will have decreased to a safe level by the time you arrive home after the scan. However, some investigations need more specific restrictions with regards to contact with young children and these will be explained.

- If you are afraid of needles you can ask for Ametop cream/spray. This is a local anaesthetic, which is placed on the skin half an hour before the injection. **Please call the Nuclear Medicine Department if you would like this.**
- If you are pregnant or breastfeeding, please contact the department as soon as possible to find out if you can have this test. Generally, nuclear medicine tests are not carried out on pregnant women unless absolutely necessary and then the dose of radioactivity will probably be reduced.
